# Two Comprehensive Liquid Chromatography High-Resolution Mass Spectrometry (UPLC-MS/MS) Multi-Methods for Real-Time Therapeutic Drug Monitoring (TDM) of Five Novel Beta-Lactams and of Fosfomycin Administered by Continuous Infusion

**DOI:** 10.3390/pharmaceutics18010091

**Published:** 2026-01-10

**Authors:** Ilaria Trozzi, Beatrice Giorgi, Riccardo De Paola, Milo Gatti, Federico Pea

**Affiliations:** 1 Clinical Pharmacology Unit, Department for Integrated Infectious Risk Management, IRCCS Azienda Ospedaliero-Universitaria di Bologna, 40138 Bologna, Italy; ilaria.trozzi@aosp.bo.it (I.T.); beatrice.giorgi@aosp.bo.it (B.G.); milo.gatti2@unibo.it (M.G.); 2 Specialisation School of Clinical Pharmacology and Toxicology, Alma Mater Studiorum Università di Bologna, 40138 Bologna, Italy; riccardo.depaola@studio.unibo.it; 3 Department of Medical and Surgical Sciences, Alma Mater Studiorum University of Bologna, 40138 Bologna, Italy

**Keywords:** β-lactam antibiotics, β-lactamase inhibitors, therapeutic drug monitoring (TDM), UPLC-qTOF-MS/MS, high resolution mass spectrometry

## Abstract

**Background/Objectives**: Therapeutic drug monitoring (TDM) of β-lactams (BL), BL/β-lactamase inhibitor (BLI) combinations (BL/BLIc), and of fosfomycin may play a key role in optimizing antimicrobial therapy and in preventing resistance development, especially when used by continuous infusion in critically ill or immunocompromised patients. Unfortunately, analytical methods for simultaneously quantifying multiple BL/BLIc in plasma are still lacking. **Methods**: The aim of this study was to develop and validate two rapid, sensitive, and accurate UPLC–qTOF–MS/MS methods for the simultaneous quantification of five novel β-lactam or β-lactam/β-lactamase inhibitor combinations (ceftolozane/tazobactam, ceftazidime/avibactam, meropenem/vaborbactam, cefiderocol, and ceftobiprole) along with fosfomycin. Methods: Human plasma samples were prepared by protein precipitation using methanol containing isotopically labeled internal standards. Chromatographic separation was achieved within 10–12 min using two Agilent Poroshell columns (EC-C18 and PFP) under positive and negative electrospray ionization modes. The method was validated according to the EMA guidelines by assessing selectivity, linearity, precision, accuracy, matrix effects, extraction recovery, and stability. **Results**: The methods exhibited excellent linearity (R^2^ ≥ 0.998) across the calibration ranges for all of the analytes (1.56–500 µg/mL), with limits of quantification ranging from 1.56 to 15.62 µg/mL. Intra- and inter-day precision and accuracy were always within ±15%. Extraction recovery always exceeded 92%, and the matrix effects were effectively corrected through isotopic internal standards. No carry-over or isobaric interferences were observed. All the analytes were stable for up to five days at 4 °C, but the BL and BL/BLIc stability was affected by multiple freeze–thaw cycles. **Conclusions**: These UPLC-qTOF-MS/MS multi-analyte methods enabled a simultaneous, reliable quantification in plasma of five novel beta-lactams and of fosfomycin. Robustness, high throughput, and sensitivity make these multi-methods feasible for real-time TDM, supporting personalized antimicrobial dosing and improved therapeutic outcomes in patients with severe or multidrug-resistant infections.

## 1. Introduction

Hospital-acquired infections represent a major healthcare issue for critically ill patients [1]. *E. coli*, *K. pneumoniae*, *P. aeruginosa*, and *A. baumannii* account for the vast majority of causative pathogens among the Gram-negatives, whereas *S. aureus* is the most frequent one among the Gram-positives. Beta-lactams (BL) and/or beta-lactam/β-lactamase inhibitor combinations (BL/BLIc) have represented for several decades the backbone of treatment of in-hospital bacterial infections [2]. Unfortunately, the extensive use of traditional BL and BL/BLIc over time has favored the selection of pathogens expressing multidrug resistance (MDR) against these agents. Consequently, in recent years, some novel BL and BL/BLIc have been introduced in the therapeutic armamentarium for counteracting the increasing prevalence of carbapenem-resistant Gram-negatives or methicillin-resistant Staphylococci, namely the most challenging etiological agents of severe infections affecting critically ill patients with a complex case mix. In this scenario, Ceftolozane/Tazobactam, Ceftazidime/Avibactam, Meropenem/Vaborbactam, and the siderophore antibiotic Cefiderocol are the most clinically relevant ones for treating infections caused by difficult-to-treat (DTR) *Pseudomonas aeruginosa*, carbapenemase-producing *Enterobacterales*, and carbapenem-resistant *Acinetobacter baumannii* [3,4]. Ceftobiprole is a fifth-generation cephalosporin that is active against methicillin-resistant *Staphylococcus aureus* (MRSA) [5]. Interestingly, fosfomycin, a phosphonic acid derivative discovered over four decades ago, has recently gained renewed interest as an agent that is active against both MDR-Gram-negatives and MDR-Gram-positives, to be used in combination therapy with novel BL or BL/BLIc [6]. Targeted antibiotic therapy based on therapeutic drug monitoring (TDM) is nowadays considered fundamental for optimizing the pharmacokinetic/pharmacodynamic (PK/PD) target attainment of antibiotic therapy among critically ill or immunocompromised patients with severe bacterial infections [7]. This approach may enhance clinical efficacy while simultaneously reducing the risk of resistance development [7]. All of these antibiotics exhibit a time-dependent antibacterial activity, meaning that they may benefit from continuous infusion (CI) administration [8,9,10,11]. CI is the administration mode granting, under the same daily dose, the highest likelihood of attaining the so-called aggressive PK/PD target attainment of BL, namely concentrations persisting four-fold above the MIC of the pathogen for the entire dosing interval [12,13,14,15,16]. Nowadays, this approach is recommended for maximizing the efficacy of BL in the treatment of infections in critically ill patients [17,18,19]. At our center, real-time TDM-based personalization of antibiotic therapy has been standard practice for five years. Unfortunately, the currently available methods are based on detecting one antibiotic at a time, so the simultaneous TDM application in routine clinical practice of all of these agents in real-time is quite complex and time-consuming [20,21,22]. Consequently, developing all-in-one analytical methods that are comprehensive of all of these antibiotics in human plasma would be very helpful for properly dealing with real-time TDM-guided optimization of antibiotic treatment by CI. In recent years, several methods describing the simultaneous quantification of multiple antibiotics have been published [23,24,25,26]. However, none of these specifically focused on innovative BL, BL/BLIc, and fosfomycin administered to critically ill patients. High-resolution techniques, such as UPLC-MS/MS coupled with quadrupole time-of-flight mass spectrometry (q-TOF), may represent a promising strategy for this purpose, thanks to features like high sensitivity, selectivity, and resolution capacity [27,28]. The aim of this study was to develop and validate two multi-analytical UPLC-qTOF-MS/MS methods for simultaneously applying real-time TDM of Ceftolozane/Tazobactam, Ceftazidime/Avibactam, Meropenem/Vaborbactam, Cefiderocol, Ceftobiprole, and Fosfomycin.

## 2. Materials and Methods

### 2.1. Reagents and Chemicals

Lyophilized standards of Ceftolozane, Tazobactam, Ceftazidime, Avibactam, Cefiderocol, Ceftobiprole, Meropenem, Vaborbactam, Fosfomycin, and drug-free human plasma were provided by Merck KgaA (Darmstadt, Germania). Internal standards (IS), namely [2H6]-Meropenem, [2H6]-Ceftazidime, [2H4-15N2]-Ceftolozane, [13C2-15N3]-Tazobactam, [2H8]-Cefiderocol, [15N-2H4]-Ceftobiprole, [13C5]-Avibactam, [13C3]-Fosfomycin, and [13C2-2H3]-Vaborbactam powders were purchased from Alsachim Shimadzu Chemistry & Diagnostics (Illkirch, France). Liquid chromatography–MS/MS grade reagents were purchased from Thermo-Fisher Scientific (Milan, Italy).

### 2.2. Preparation of Stock Solution, Calibrators, and Quality Control Samples

A unique stock solution containing Meropenem 200 μg/mL, Ceftazidime 200 μg/mL, Ceftolozane 200 μg/mL, Tazobactam 100 μg/mL, Cefiderocol 200 μg/mL, Ceftobiprole 100 μg/mL, Avibactam 50 μg/mL, Fosfomycin 500 μg/mL, and Vaborbactam 200 μg/mL was prepared by dissolving each analyte into Milliq water. Calibrators for the calibration curves and independent quality control (QC) samples were prepared in MilliQ water at different concentrations as serial dilutions from concentrated stock solutions, spiking drug-free plasma in each of them. Calibration points and QC samples for each drug are summarized in Table 1 and Table 2, respectively. The dynamic range of each calibration curve was based on the expected therapeutic plasma concentrations of the analyte in critically ill patients receiving treatment by CI.

A single stock solution containing 2 μg/mL [2H6]-Meropenem, 4 μg/mL [2H6]-Ceftazidime, 2 μg/mL [2H4-15N2]-Ceftolozane, 1 μg/mL [13C2-15N3]-Tazobactam, 3 μg/mL [2H8]-Cefiderocol, 3 μg/mL [15N-2H4]-Ceftobiprole, 1 μg/mL [13C5]-Avibactam, 1.5 μg/mL [13C3]—Fosfomycin, and 4 μg/mL [13C2-2H3]—Vaborbactam was prepared and used as internal standard (IS). All solvents and matrix solutions were stored at −80 °C.

### 2.3. Sample Pre-Treatment

For sample preparation, 10 µL of human plasma was added to 40 µL of ultrapure water and, subsequently, 150 µL of IS–methanol solution was added. After vortex mixing for 15 s, samples were centrifuged at 13.000 rpm for 5 min at room temperature. Following centrifugation, 100 µL of the resulting clear supernatant was transferred into an autosampler vial, 2 µL of which was injected into the LC–MS/MS system.

### 2.4. LC-MS/MS Instrumentation and Analysis

Samples were analyzed by means of a Shimadzu Nexera U-HPLC system equipped with binary pumps for mobile-phase delivery and coupled with a Quadrupole Time-of-Flight mass spectrometer X500 QTOF (AB SCIEX, Marlborough, MA, USA). Data acquisition was performed in Multiple Reaction Monitoring (MRM) mode, with electrospray ionization (ESI) operating in positive mode for β-lactams and in negative mode for β-lactamase inhibitors and fosfomycin. Separation of the different drugs was performed by using two different columns, depending on the chemical structure of the molecule, and ensuring the elution of each analyte at a specific retention time. In particular, an Agilent Poroshell 120 EC-C18 (2.1 × 50 mm, 1.9 Micron) column was used for Meropenem, Ceftazidime, Ceftolozane, Tazobactam, Cefiderocol, and Ceftobiprole; a Poroshell 120 PFP (2.1 × 50 mm, 2.7 Micron) was used for Avibactam, Fosfomycin, and Vaborbactam. Both columns were maintained at 45 °C and coupled with an autosampler set at 4 °C.

Analyte separation was achieved by using a binary pump system with a linear gradient elution from mobile-phase A [water with 0.2% formic acid, *v*/*v*] to mobile-phase B [methanol/acetonitrile 50:50 with 0.2% formic acid, *v*/*v*] at a flow rate of 0.5 mL/min (Table 3a,b).

Data acquisition was carried out in MRM mode, with electrospray ionization (ESI) operating in either the positive or negative mode. The MS/MS parameters are summarized in Table 4a,b. Chromatographic data acquisition, peak integration, and quantification were performed by using the Sciex OS 3.1.6 software (AB SCIEX, Marlborough, MA, USA).

### 2.5. Validation Procedures

The method was validated in agreement with the European Medicines Agency (EMA) guidelines for bioanalytical method development [29]. Assessments included selectivity, linearity, accuracy, precision, limit of quantification (LOQ), recovery, matrix effects, and stability.

#### 2.5.1. Selectivity and Carry-Over

Thirty plasma samples were analyzed to test the absence of response from endogenous or exogenous components in the matrix at the retention time of each drug under investigation. The carry-over was assessed by injecting a blank sample immediately after finishing the run of the highest calibrator (calibrator 6), and it was considered negligible whenever the signal intensity was less than 20% of that of the Low Limit of Quantification (LLOQ), as recommended by the EMA guidelines [29]. 

#### 2.5.2. Linearity and Limit of Quantification (LOQ)

As recommended by the EMA guidelines, 1/x weighing linear regression was used for building the calibration curves. These were defined as linear whenever the linear regression coefficient (R^2^) was ≥0.998. The LOQ of the methods was defined as the lowest concentration, covered by the dynamic range, showing a signal-to-noise ratio (S/N) higher than 10, and corresponded to the calibrator number 1 of each analyte.

#### 2.5.3. Precision and Accuracy

Precision (expressed as mean CV%) and accuracy (expressed as mean BIAS%) were determined by analyzing the LOQ, LQC, MQC, and HQC five times within the same day (intra-day) across three separate inter-day analytical sessions.

#### 2.5.4. Matrix Effect and Extraction Recovery Percent

Matrix effect (ME) and extraction recovery (ER) were evaluated at the three QC levels (LQC, MQC, and HQC) by using plasma samples collected from ten different patients to properly address inter-individual variability in matrix composition. The matrix effect was evaluated quantitatively by comparing plasma samples spiked with the analyte with water samples spiked with the same analyte. The analyte ratios measured in the corresponding plasma and water samples were compared, and the average and coefficient of variation (CV) across six different batches were calculated. The extraction recovery was assessed by comparing the plasma extracts spiked with the analytes and with the IS with those spiked with only the IS. The compensated matrix effect was determined by calculating the area ratio between the analyte and the respective IS and should have been <15%.

#### 2.5.5. Stability

Stability of the antibiotics in human plasma, as well as that of the extracted samples, was evaluated at different concentrations of the calibration range (LQC, MQC, and HQC) under different storage conditions. According to laboratory requirements and routine practice, the following conditions were tested:

1. Extracts kept at 4 °C over 24 h, for 5 d;
2. Extracts after three complete freeze and thaw cycles from −80 °C to 25 °C.

Sample concentrations measured before and after storage were compared to the nominal values, and stability was confirmed whenever the measured concentration ranged within ±15% of the nominal concentration.

### 2.6. Application to Clinical Samples

The developed LC-MS/MS multi-method was validated by assessing the plasma steady-state concentrations of the different antibiotics in plasma samples collected from 30 hospitalized patients receiving treatment by CI with one of the investigated novel BL or BL/BLIc as monotherapy or as combination therapy with Fosfomycin. Samples were processed immediately after collection. The study was carried out in accordance with the principles of the Declaration of Helsinki and received approval from the Ethics Committee of the Azienda Ospedaliero-Universitaria di Bologna (Approval No. 442/2021/Oss/AOUBo, 28 June 2021). Samples were collected during routine clinical practice and were used for validating previously single analytical methods [20,21,22].

## 3. Results

### 3.1. Analytical Performance

By examining the MS/MS fragmentation pattern spectra of all of the analytes, single-charge positive and negative mass transitions were selected, and the parameters were optimized to obtain the best MRM (Multiple Reaction Monitoring) signals, as shown in Table 5.

**Figure 1:**
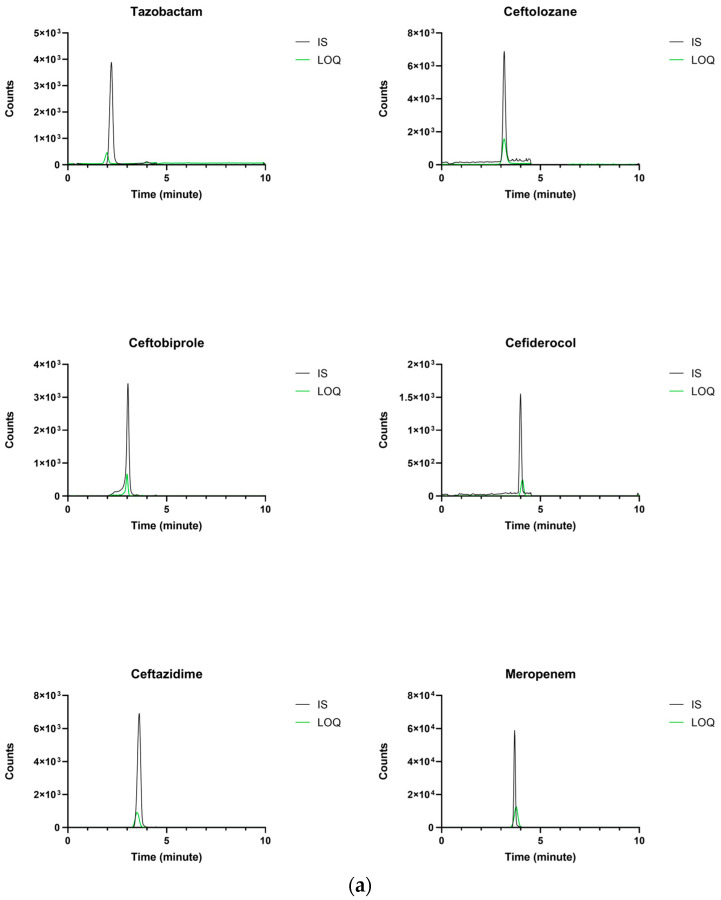

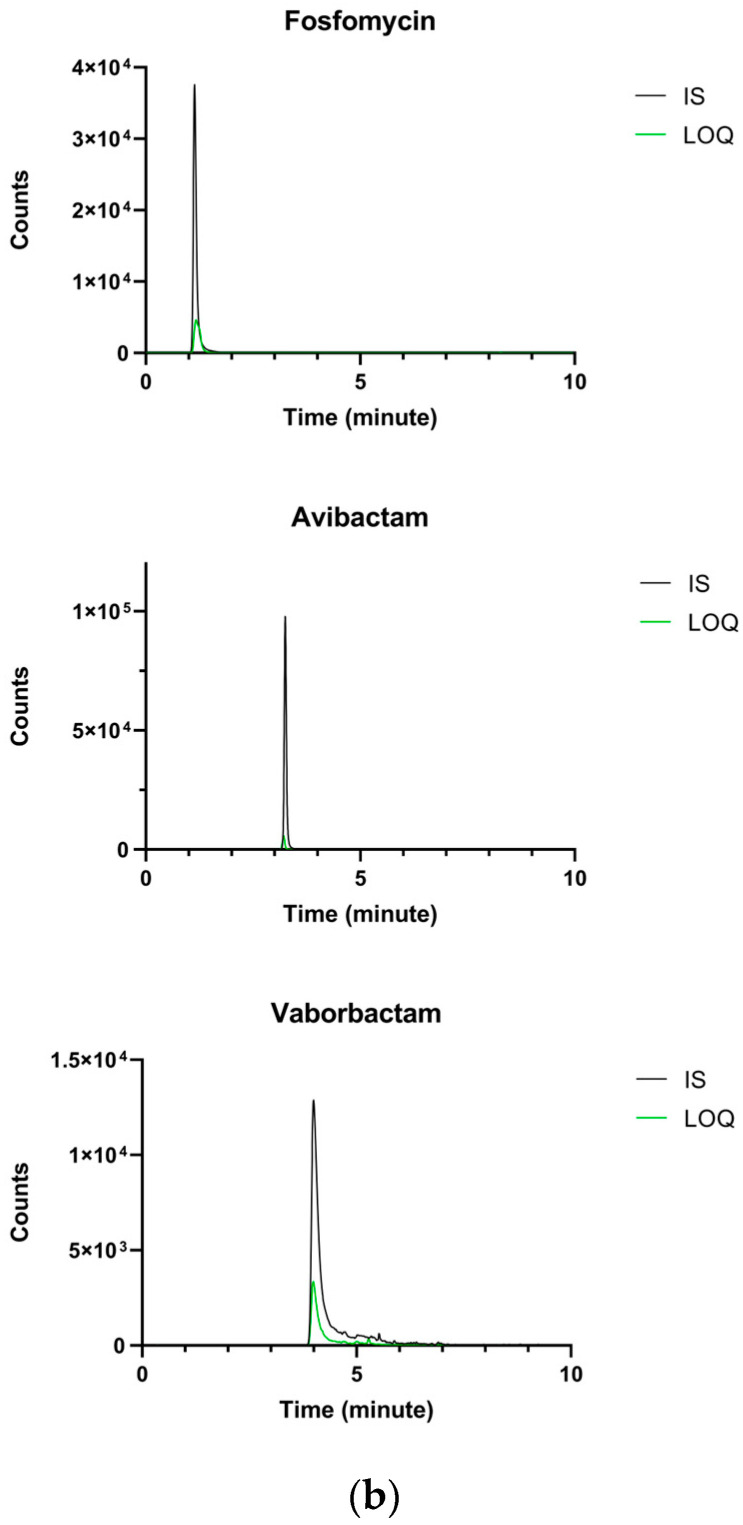
(**a**) Overlayed MRM chromatograms at the MDL value of Meropenem, Ceftazidime, Ceftolozane, Tazobactam, Cefiderocol, Ceftobiprole (green color), and of the respective IS (black color). (**b**) Overlayed MRM chromatograms at the MDL value of Vaborbactam, Fosfomycin, Avibactam (green color), and of the respective IS (black).

**Figure 2:**
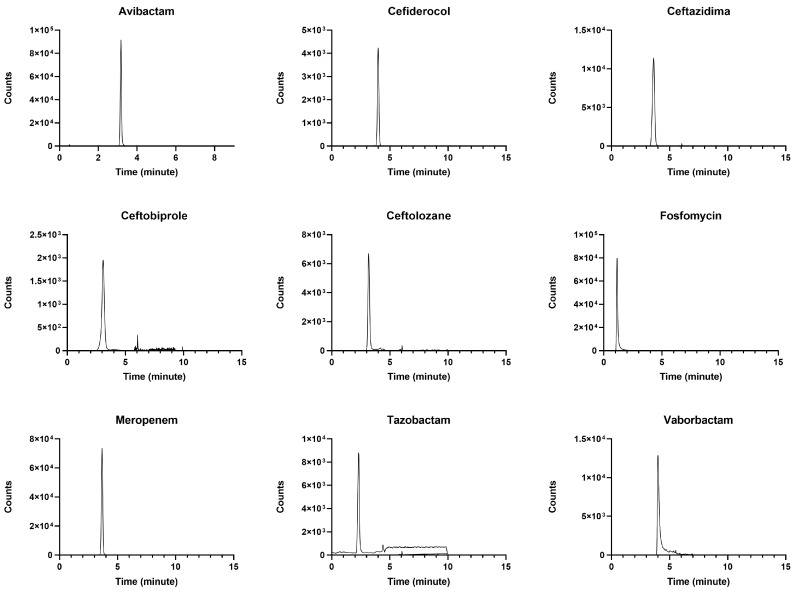
MRM chromatograms of real patient samples showing good peak shape and resolution of specific peaks for each analyte.

The selected LC methods allowed for clear, high-quality chromatographic peaks, as depicted in Figure 1a,b, with retention times absolutely constant and reproducible. This may support effective column reconditioning throughout the analyses. The MRM chromatogram of the blank sample prepared with IS-methanol solution showed a resolved peak and was overimposable to that of the corresponding analyte related at the expected retention time. Chromatograms of real patient samples (Figure 2) showed sharp peak shapes and high resolution with no isobaric interference, further confirming the selectivity of the applied MRM transitions. This finding is consistent with both the specificity of the MRM transitions and the purity of the internal standard solutions. The signal-to-noise (S/N) ratios at the LLOD were notably high for all of the analytes (namely (S/N) = 2181.3 for Meropenem, (S/N) = 837.5 for Ceftazidime, (S/N) = 225.6 for Ceftolozane, (S/N) = 281.9 for Cefiderocol, (S/N) = 797.4 for Vaborbactam, (S/N) = 142.1 for Tazobactam, (S/N) = 276.8 for Ceftobiprole, (S/N) = 636.3 for Avibactam, and (S/N) = 1405.8 for Fosfomycin), supporting the high sensitivity of the method.

### 3.2. Method Validation

The methods validated according to the EMA guidelines showed excellent selectivity and specificity with no interfering peaks at the optimized chromatographic conditions. Thanks to the small injection volume (2 µL), carry-over was absent. The linear regression fit of all the calibration curves was excellent (with R^2^ ≥ 0.998) (Table 6).

All back-calculated values did not differ from ±15% of the theoretical value. The results of the intra- and inter-assay precision (CV%) and accuracy (Bias %) were always within the acceptable ranges, as shown in Table 7.

The percent matrix effect (%ME), IS—normalized matrix effect, and percent extraction recovery yield (%ER) of the LQC, MQC, and HQC levels of the different analytes are summarized in Table 8. Positive %ME with signal enhancement and increasing methods sensitivity was observed for Ceftazidime, Avibactam, Ceftolozane, Tazobactam, and Cefiderocol at all of the tested concentrations. Conversely, negative %ME with signal suppression was observed for Meropenem, Vaborbactam, Ceftobiprole, and Fosfomycin. Signal normalization by means of the relevant IS was then applied to properly meet the EMA validation criteria for matrix effect. The %ER approximated 100% for all of the analytes at all of the three tested concentrations.

### 3.3. Stability

The results of the stability tests are summarized in Table 9. The stability profiles in plasma samples of the LQC, MQC, and HQC kept at 4 °C over 24 h for 5 days, and after three complete freeze and thaw cycles from −80 °C to 25 °C, were quite different from one molecule to another. Specifically, all the molecules were stable when the LQC, MQC, and HQC were kept at 4 °C for over 24 h for 5 days. Conversely, Tazobactam, Avibactam, and Fosfomycin were stable after all of the three complete freeze and thaw cycles, whereas Ceftobiprole, Ceftazidime, and Ceftolozane were stable after only two, Cefiderocol and Vaborbactam after only one, and Meropenem had borderline stability for one cycle.

### 3.4. Clinical Validation

**Figure 3:**
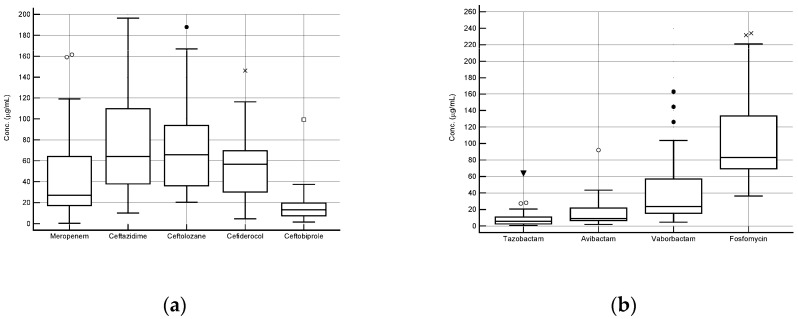
Box and whisker plots of the steady-state concentrations of β-lactams (**a**) and of β-lactamase inhibitors and fosfomycin (**b**) measured in the plasma of 30 hospitalized patients. The symbols such as dots, crosses and triangles in the image represent outlier values for each analyte.

Box and whisker plots of the steady-state plasma concentrations of the different antibiotics observed in 30 critically ill patients undergoing real-time TDM are shown in Figure 3. Overall, the measured concentrations fell within the calibration range selected for each agent (2.6–161.5 μg/mL for Meropenem, 20.4–187.9 μg/mL for Ceftolozane, 0.4–63.7 μg/mL for Tazobactam, 10.1–196.5 μg/mL for Ceftazidime, 4.7–146.3 μg/mL for Cefiderocol, 1.6–99.3 μg/mL for Ceftobiprole, 4.6–162.9 μg/mL for Vaborbactam, 1.9–92 μg/mL for Avibactam, and 36.4–291.1 μg/mL for Fosfomycin). The chromatograms showed sharp peak shapes and high resolution, even in the presence of low concentration values for all of the different agents. No isobaric interference was detected.

## 4. Discussion

The methods developed by means of the UPLC-qTOF-MS/MS technique in this study were revealed to be reliable, highly sensitive, and specific for simultaneously quantifying five different novel BL and/or BL/BLIc and fosfomycin in human plasma. Importantly, it should be highlighted that even if the retention times of some agents could be quite similar, this does not represent a real issue when applying these methods for routine TDM purposes in clinical practice. In fact, meropenem/vaborbactam, ceftazidime/avibactam, ceftolozane/tazobactam, and cefiderocol are never administered in combination therapy to the same patient. Conversely, the opportunity of having 6-in-1 and 3-in-1 methods to be applied in real-time may allow an efficacious response to a growing clinical need. Indeed, our novel approach allowed measuring plasma concentrations of the different agents within a turnaround time of approximatively 3 h after sample delivery. This may allow for prompt dosing optimization within the same day. Our two multi-method approaches may have two main advantages compared to the conventional methods of individually quantifying each of the analytes under investigation [20,21,22,30,31,32], namely enhanced efficiency and rapidity, since only two rather than six chromatographic runs are needed. Additionally, these methods may provide better consistency and comparability of data, since all analytes undergo similar pre-analytical processing. From a clinical standpoint, having shorter turnaround times make possible to make timely dose adjustments and process a much higher number of different samples within the same timeframe. The TDM-guided approach is acquiring a more and more crucial role in both maximizing the efficacy of these antibiotics and minimizing the risk of resistance development against them [33,34]. The possibility of analyzing multiple molecules simultaneously saves both time and cost, and may favor the feasibility of such an approach in daily clinical practice. It is worth mentioning that adopting isotopically labeled internal standards made it possible to effectively correct for plasma matrix variability, namely an especially relevant issue in heterogeneous populations like the critically ill patients [25]. This improved the robustness of the methods, which are stable and reliable even under storage and handling conditions typical of clinical laboratories. In this regard, it should not be overlooked that stability may be a critical concern for some of the investigated drugs [35,36]. Importantly, all the molecules were stable for up to 5 days when kept at +4 °C. This means that since the autosampler extracts are kept at 4 °C over time, it may be feasible to reliably reanalyze the sample extracts several times. Conversely, attention should be paid to frozen samples. Only three out of nine molecules were stable for all of the three freeze and thaw cycles (Tazobactam, Avibactam, and Fosfomycin), other three agents for two (Ceftazidime, Ceftolozane, and Ceftobiprole), two agents for one (Cefiderocol and Vaborbactam), and the last, Meropenem, was borderline for one. This issue was just previously found in other methods testing single drugs [22,24]. Stability data indicate that most analytes may withstand up to two or three freeze–thaw cycles, thus allowing the delivery, storage, and reprocessing of samples. In case of unstable compounds exhibiting a withstand of a maximum of one freeze–thaw cycle, namely Meropenem/Vaborbactam, blood samples should be kept at 4 °C during delivery, and processing of fresh plasma samples is recommended, preferably. However, if samples were collected in a remote hospital, blood samples should be centrifuged locally, and the supernatant should be separated promptly and then stored in dry ice during delivery up to processing, so that they may undergo a maximum of one freeze–thaw cycle. A valuable advantage of qTOF system-based methods compared to those traditionally based on liquid chromatography with UV detection or low-resolution mass spectrometry was the high resolution and accuracy in both identifying and quantifying the analytes. This may greatly reduce the risk of detecting interfering signals and/or false positives. An additional advantage is that of being versatile, namely, it could be implemented in the future with other additional molecules according to clinical and research needs. Limitations of the developed methods should be acknowledged. We recognize that these methods could be feasible for real-time TDM only in patients receiving the investigated antibiotics by CI and not by intermittent infusion. Specifically regarding this latter mode of administration, it should not be overlooked that the LOQs of avibactam and tazobactam set in our methods could not be low enough for reliably measuring the trough levels, especially in patients experiencing augmented renal clearance. In fact, the linear dynamic ranges of the calibration curves were set based on the plasma therapeutic ranges expected in critically ill patients treated by CI. Notably, this approach is consistent with recent recommendations issued by the French Intensive Care Society focusing on the proper use of novel BL/BLIc and cefiderocol in critically ill patients [37]. Indeed, administering these agents by prolonged and/or CI was strongly suggested for maximizing the likelihood of attaining aggressive PK/PD targets of novel BL and/or BL/BLIc in critically ill patients [37]. It should be noted that administering these agents by CI may greatly reduce the risk of having Css of BLI below the set LOQs. To this regard, our recent study conducted among 263 patients receiving a TDM-guided therapy with CI ceftazidime/avibactam and ceftolozane/tazobactam [38], showed that the median avibactam and tazobactam Css were as high as 10.25 mg/L (IQR 5.33–16.78 mg/L) and 10.6 mg/L (IQR 6.5–17.6 mg/L), respectively, and that only 1.7% and 3.9% of avibactam and tazobactam concentrations were below the set LOQs. Furthermore, implementing a TDM-guided strategy for maximizing PK/PD target attainment of BL in critically ill patients has been strongly recommended by different guidance [19,37,39]. A recent meta-analysis including eleven randomized controlled trials and observational studies reported that implementing a TDM-guided approach for BL was significantly associated with a higher clinical cure rate, lower microbiological failure rate, and higher likelihood of aggressive PK/PD target attainment in critically ill patients [40]. Notably, a recent pre–post quasi-experimental study demonstrated that a multidisciplinary strategy targeting aggressive joint PK/PD targets of CI TDM-guided ceftazidime/avibactam therapy was effective in both improving clinical and microbiological outcomes of KPC-producing *Klebsiella pneumoniae* infections and reducing resistance development to ceftazidime/avibactam [41]. However, we acknowledge that significant barriers may limit a widespread implementation of our approach, with user-friendly analytical methods of novel BL/BLI combinations often being unavailable and frequently lacking accurate expertise for proper interpretation of TDM findings [19].

In summary, the developed UPLC-qTOF-MS/MS methods may represent a valuable tool for the simultaneous real-time TDM of a wide range of novel BL and/or BL/BLIc delivered by CI. This approach, by personalizing antimicrobial therapies, may be helpful in counteracting the growing threat of bacterial resistance and in optimizing treatment efficacy while reducing the neurotoxicity risk associated with peak levels that are too high [23].

## 5. Conclusions

In conclusion, two fast, sensitive, and accurate UPLC-qTOF MS/MS methods have been developed and validated for quantifying Meropenem/Vaborbactam, Ceftazidime/Avibactam, Ceftolozane/Tazobactam, Cefiderocol, Ceftobiprole, and Fosfomycin in human plasma samples. Thanks to high performance and rapid execution times, the assays provide a robust bioanalytical platform for applying real-time TDM-guided dosing adjustment of these antibiotics in the clinical setting.

## Figures and Tables

**Table 1:** Dynamic ranges of the calibration curve for each analyte.

Analyte	Calibrator 1 (μg/mL)	Calibrator 2 (μg/mL)	Calibrator 3 (μg/mL)	Calibrator 4 (μg/mL)	Calibrator 5 (μg/mL)	Calibrator 6 (μg/mL)
Meropenem	6.25	12.5	25	50	100	200
Ceftazidime	6.25	12.5	25	50	100	200
Ceftolozane	6.25	12.5	25	50	100	200
Tazobactam	3.13	6.25	12.5	25	50	100
Cefiderocol	6.25	12.5	25	50	100	200
Ceftobiprole	3.13	6.25	12.5	25	50	100
Avibactam	1.56	3.13	6.25	12.5	25	50
Fosfomycin	15.62	31.25	62.5	125	250	500
Vaborbactam	6.25	12.5	25	50	100	200

**Table 2:** Quality control (QC) sample concentrations expressed in μg/mL.

Analyte	LOQ (Limit of Quantification)	LQC (Low QC)	MQC (Medium QC)	HQC (High QC)
Meropenem	6.25	10	25	50
Ceftazidime	6.25	10	25	50
Ceftolozane	6.25	10	25	50
Tazobactam	3.13	5	12.5	25
Cefiderocol	6.25	10	25	50
Ceftobiprole	3.13	5	12.5	25
Avibactam	1.56	2	6.25	12.5
Fosfomycin	15.62	20	62.5	125
Vaborbactam	6.25	10	25	50

**Table 3:** (**a**) UPLC method for the 120 EC-C18 column. (**b**) UPLC method for the 120 PFP column.

(**a**)			
**Time (min.)**	**A (%)**	**B (%)**	**Flow (mL/min.)**
0.00	98	2	0.5
0.20	98	2	0.5
4.00	40	60	0.5
4.10	2	98	0.5
5.50	2	98	0.5
5.60	98	2	0.5
10.0	98	2	0.5
(**b**)			
**Time (min.)**	**A (%)**	**B (%)**	**Flow (mL/min.)**
0.00	98	2	0.5
0.10	98	2	0.5
5.00	0	100	0.5
7.00	0	100	0.5
7.10	98	2	0.5
12.0	98	2	0.5

**Table 4:** (**a**) MS/MS parameters for positive polarization. (**b**) MS/MS parameters for negative polarization.

(**a**)						
**Ion source gas 1** **pressure**	**Ion source gas 1** **Pressure**	**Curtain gas pressure**	**CAD gas**	**Gas** **temperature**	**Polarity**	**Spray voltage**
45 psi	55 psi	35 psi	7	550 °C	Positive	5500 V
**TOF start-stop mass**	**Accumulation time**	**Declustering potential**	**DP spread**	**Collision energy (CE)**	**CE spread**	
250–850 Da	0.25 s	40 V	0 V	5 V	0 V	
(**b**)						
**Ion source gas 1** **pressure**	**Ion source gas 1** **Pressure**	**Curtain gas pressure**	**CAD gas**	**Gas** **temperature**	**Polarity**	**Spray voltage**
40 psi	45 psi	35 psi	7	450 °C	Negative	−4500 V
**TOF start-stop mass**	**Accumulation time**	**Declustering potential**	**DP spread**	**Collision energy (CE)**	**CE spread**	
100–400 Da	0.1 s	−40 V	0 V	−5 V	0 V	

**Table 5:** MRM transition parameters used for each analyte and its respective IS (internal standard) acquisition.

Analyte	Retention Time (min.)	Precursor Ion—Quantifier (*m*/*z*)	Product Ion—Qualifier (*m*/*z*)	Accumulation Time (ms)	DP (V)	CE (V)
Meropenem	3.7	384.00000	141.10260	50	27	20
[2H6]-Meropenem	3.7	390.00000	147.14010	50	27	20
Ceftazidime	3.6	547.50000	167.02770	50	40	35
[2H6]-Ceftazidime	3.6	553.50000	167.02820	50	40	35
Ceftolozane	3.2	334.50000	139.06190	50	20	30
[2H4-15N2]-Ceftolozane	3.2	337.50000	139.06240	50	20	30
Tazobactam	1.9	301.28900	122.06100	50	20	25
[13C2-15N3]-Tazobactam	1.9	306.28900	122.06040	50	20	25
Cefiderocol	3.9	752.20000	285.10040	50	20	30
[2H8]-Cefiderocol	3.9	760.20000	293.15040	50	20	30
Ceftobiprole	3.1	535.10000	203.11860	50	40	40
[15N-2H4]-Ceftobiprole	3.1	540.10000	208.14060	50	40	40
Avibactam	2.7	264.03080	95.95280	50	−40	−30
[13C5]-Avibactam	2.7	269.04730	95.95260	50	−40	−30
Fosfomycin	1.02	137.00160	78.95970	50	−40	−20
[13C3]-Fosfomycin	1.02	301.0000	78.95960	50	−40	−20
Vaborbactam	3.86	296.07790	234.07730	50	−40	−25
[13C2-2H3]-Vaborbactam	3.86	301.10090	239.10050	50	−40	−25

**Table 6:** Linearity ranges, regression equations, and coefficients of determination (R^2^) relative to the calibration curves, obtained with a 1/x weighting linear regression.

Analyte	Dynamic Range (µg/mL)	Regression Equation	R^2^
Meropenem	6.25–200	y = 0.041 (± 0.006)x + 0.424 (± 0.003)	0.998
Ceftazidime	6.25–200	y = 0.040 (± 0.004)x − 0.381 (± 0.003)	0.999
Ceftolozane	6.25–200	y = 0.031 (± 0.003)x + 0.232 (± 0.002)	0.998
Tazobactam	3.13–100	y = 0.249 (± 0.005)x + 0.669 (± 0.002)	0.999
Cefiderocol	6.25–200	y = 0.046 (± 0.005)x − 0.142 (± 0.003)	0.999
Ceftobiprole	3.13–100	y = 0.062 (± 0.007)x + 0.045 (± 0.004)	0.999
Avibactam	1.56–50	y = 0.043 (± 0.002)x + 0.011 (± 0.002)	0.999
Fosfomycin	15.62–500	y = 0.125 (± 0.003)x + 1.493 (± 0.004)	0.998
Vaborbactam	6.25–200	y = 0.089 (± 0.004)x + 0.047 (± 0.002)	0.999

**Table 7:** Intra-day and inter-day average (Avg) precision and accuracy were evaluated at four concentration levels (LOQ, LQC, MQC, and HQC) by analyzing five times each level within the same day (intra-day) across three separate daily analytical runs (inter-day).

QC Levels			Intraday (n = 5)			Inter—Day		
Sample	Analyte	Nominal Conc. (μg/mL)	Avg Conc. (μg/mL)	Avg Precision (CV%)	Avg Accuracy (Bias %)	Avg Conc. (μg/mL)	Avg Precision (CV%)	Avg Accuracy (Bias %)
**LOQ**	Meropenem	6.25	5.78	7.28	−7.52	6.00	7.26	−4.00
	Ceftazidime	6.25	6.72	11.90	7.53	6.73	10.11	7.73
	Ceftolozane	6.25	5.76	11.00	−7.84	6.00	7.64	−4.00
	Tazobactam	3.13	2.94	13.10	−5.92	3.03	13.70	−2.93
	Cefiderocol	6.25	6.56	6.52	4.96	6.60	6.06	5.60
	Ceftobiprole	3.13	2.78	9.98	−11.00	2.93	8.58	−6.13
	Avibactam	1.56	1.52	15.70	−2.56	1.40	14.30	−10.30
	Fosfomycin	15.62	15.48	5.54	−0.93	14.30	10.20	−8.27
	Vaborbactam	6.25	6.14	7.15	−1.76	6.13	9.96	−1.87
**LQC**	Meropenem	10.00	10.08	0.68	0.80	10.30	6.37	3.00
	Ceftazidime	10.00	9.90	0.59	−1.00	10.13	6.42	1.33
	Ceftolozane	10.00	10.12	0.67	1.20	10.33	6.44	3.33
	Tazobactam	5.00	4.90	0.64	−2.00	5.03	13.95	0.67
	Cefiderocol	10.00	9.96	0.49	−0.40	9.97	4.18	−0.33
	Ceftobiprole	5.00	5.08	0.58	1.60	5.33	9.62	6.67
	Avibactam	2.00	2.02	0.26	1.00	2.03	10.24	1.67
	Fosfomycin	20.00	19.94	0.88	−0.30	20.07	5.67	0.33
	Vaborbactam	10.00	10.06	0.79	0.60	10.33	5.83	3.33
**MQC**	Meropenem	25.00	24.62	2.56	−1.52	24.87	2.68	−0.53
	Ceftazidime	25.00	25.36	2.68	1.44	25.23	3.23	0.93
	Ceftolozane	25.00	24.50	2.72	−2.00	24.87	2.68	−0.53
	Tazobactam	12.50	12.38	4.36	−0.96	12.70	3.43	1.60
	Cefiderocol	25.00	24.98	3.60	−0.08	25.40	3.80	1.60
	Ceftobiprole	12.50	11.94	6.74	−4.48	12.27	7.70	−1.87
	Avibactam	6.25	5.96	6.77	−4.64	6.03	9.99	−3.47
	Fosfomycin	62.50	61.96	1.59	−0.86	62.17	1.39	−0.53
	Vaborbactam	25.00	24.78	3.36	−0.88	24.77	3.66	−0.93
**HQC**	Meropenem	50.00	49.60	1.20	−0.80	49.50	1.65	−1.00
	Ceftazidime	50.00	50.52	0.91	1.04	50.37	1.29	0.73
	Ceftolozane	50.00	49.42	1.43	−1.16	49.70	1.61	−0.60
	Tazobactam	25.00	24.54	1.95	−1.84	24.97	2.84	−0.13
	Cefiderocol	50.00	50.40	0.92	0.80	50.37	1.19	0.73
	Ceftobiprole	25.00	24.50	2.48	−2.00	25.03	2.94	0.13
	Avibactam	12.50	12.38	4.62	−0.96	12.53	5.88	0.27
	Fosfomycin	125.00	125.60	1.03	0.48	126.00	1.31	0.80
	Vaborbactam	50.00	49.60	1.31	−0.08	49.70	1.41	−0.6

**Table 8:** Average (Avg) matrix effect (ME%), IS—normalized matrix effect, and extraction recovery (ER%) determined at different concentration levels for each analyte.

Quality Control Levels	Analyte	N° Replicates	Avg Matrix Effect (%)	Avg IS-Normalized Matrix Effect (%)	Avg Extraction Recovery (%)
**LQC**	Meropenem	30	−1.68	0.42	93.34
	Ceftazidime	30	45.30	−0.26	99.89
	Ceftolozane	30	8.72	3.06	95.97
	Tazobactam	30	38.9	0.48	98.72
	Cefiderocol	30	7.37	−0.56	99.96
	Ceftobiprole	30	−18.40	−0.32	97.60
	Avibactam	30	27.98	0.96	96.02
	Fosfomycin	30	−14.40	−0.23	98.78
	Vaborbactam	30	−18.11	−4.31	98.20
**MQC**	Meropenem	30	−5.24	1.75	99.00
	Ceftazidime	30	44.62	−0.25	97.62
	Ceftolozane	30	8.82	3.64	97.72
	Tazobactam	30	37.78	0.47	96.28
	Cefiderocol	30	10.22	−0.41	98.77
	Ceftobiprole	30	−14.33	−0.24	98.06
	Avibactam	30	23.85	−0.99	97.51
	Fosfomycin	30	−17.33	−0.27	92.94
	Vaborbactam	30	−19.03	−3.44	95.86
**HQC**	Meropenem	30	−3.96	−0.71	92.75
	Ceftazidime	30	40.41	−0.21	97.22
	Ceftolozane	30	2.03	−1.04	98.51
	Tazobactam	30	28.31	0.35	96.68
	Cefiderocol	30	10.95	−1.60	95.08
	Ceftobiprole	30	−38.22	−0.63	99.45
	Avibactam	30	14.63	−0.79	97.52
	Fosfomycin	30	−30.12	−0.46	96.19
	Vaborbactam	30	−20.68	−4.01	98.54

**Table 9:** Stability of the analytes at different storage conditions.

Quality Control Levels	Analyte	Sample	Tested Conditions	Average Accuracy (Bias%)
**LQC**	Meropenem	extract	Autosampler, day 1	13.00
			Autosampler, day 5	−2.00
		plasma	Freeze–thaw stability 1 cycle	−15.70
			Freeze–thaw stability 2 cycle	−36.10
			Freeze–thaw stability 3 cycle	−65.40
	Ceftazidime	extract	Autosampler, day 1	10.00
			Autosampler, day 5	7.00
		plasma	Freeze–thaw stability 1 cycle	−4.30
			Freeze–thaw stability 2 cycle	−7.80
			Freeze–thaw stability 3 cycle	−15.30
	Ceftolozane	extract	Autosampler, day 1	10.60
			Autosampler, day 5	9.00
		plasma	Freeze–thaw stability 1 cycle	−2.10
			Freeze–thaw stability 2 cycle	−6.80
			Freeze–thaw stability 3 cycle	−15.50
	Tazobactam	extract	Autosampler, day 1	5.50
			Autosampler, day 5	8.00
		plasma	Freeze–thaw stability 1 cycle	−1.20
			Freeze–thaw stability 2 cycle	−6.20
			Freeze–thaw stability 3 cycle	−14.60
	Cefiderocol	extract	Autosampler, day 1	7.00
			Autosampler, day 5	8.00
		plasma	Freeze–thaw stability 1 cycle	−14.90
			Freeze–thaw stability 2 cycle	−35.30
			Freeze–thaw stability 3 cycle	−71.50
	Ceftobiprole	extract	Autosampler, day 1	2.00
			Autosampler, day 5	8.00
		plasma	Freeze–thaw stability 1 cycle	−5.10
			Freeze–thaw stability 2 cycle	−8.60
			Freeze–thaw stability 3 cycle	−14.80
	Avibactam	extract	Autosampler, day 1	−5.00
			Autosampler, day 5	10.00
		plasma	Freeze–thaw stability 1 cycle	−1.80
			Freeze–thaw stability 2 cycle	−3.80
			Freeze–thaw stability 3 cycle	−8.10
	Fosfomycin	extract	Autosampler, day 1	−3.50
			Autosampler, day 5	−2.50
		plasma	Freeze–thaw stability 1 cycle	−3.10
			Freeze–thaw stability 2 cycle	−3.40
			Freeze–thaw stability 3 cycle	−2.97
	Vaborbactam	extract	Autosampler, day 1	−3.00
			Autosampler, day 5	1.99
		plasma	Freeze–thaw stability 1 cycle	−9.10
			Freeze–thaw stability 2 cycle	−15.80
			Freeze–thaw stability 3 cycle	−2.70
**MQC**	Meropenem	extract	Autosampler, day 1	2.00
			Autosampler, day 5	5.20
		plasma	Freeze–thaw stability 1 cycle	−16.10
			Freeze–thaw stability 2 cycle	−32.00
			Freeze–thaw stability 3 cycle	−61.90
	Ceftazidime	extract	Autosampler, day 1	−8.00
			Autosampler, day 5	4.40
		plasma	Freeze–thaw stability 1 cycle	−4.80
			Freeze–thaw stability 2 cycle	−8.20
			Freeze–thaw stability 3 cycle	−16.50
	Ceftolozane	extract	Autosampler, day 1	−1.60
			Autosampler, day 5	−1.20
		plasma	Freeze–thaw stability 1 cycle	−1.80
			Freeze–thaw stability 2 cycle	−7.80
			Freeze–thaw stability 3 cycle	−15.70
	Tazobactam	extract	Autosampler, day 1	0.80
			Autosampler, day 5	−0.80
		plasma	Freeze–thaw stability 1 cycle	−2.40
			Freeze–thaw stability 2 cycle	−5.80
			Freeze–thaw stability 3 cycle	−10.90
	Cefiderocol	extract	Autosampler, day 1	−6.00
			Autosampler, day 5	−4.00
		plasma	Freeze–thaw stability 1 cycle	−14.50
			Freeze–thaw stability 2 cycle	−37.00
			Freeze–thaw stability 3 cycle	−74.10
	Ceftobiprole	extract	Autosampler, day 1	−4.00
			Autosampler, day 5	0.80
		plasma	Freeze–thaw stability 1 cycle	−4.80
			Freeze–thaw stability 2 cycle	−7.60
			Freeze–thaw stability 3 cycle	−15.40
	Avibactam	extract	Autosampler, day 1	4.00
			Autosampler, day 5	2.40
		plasma	Freeze–thaw stability 1 cycle	−2.20
			Freeze–thaw stability 2 cycle	−4.70
			Freeze–thaw stability 3 cycle	−8.50
	Fosfomycin	extract	Autosampler, day 1	3.36
			Autosampler, day 5	4.32
		plasma	Freeze–thaw stability 1 cycle	−4.20
			Freeze–thaw stability 2 cycle	−3.98
			Freeze–thaw stability 3 cycle	−3.65
	Vaborbactam	extract	Autosampler, day 1	6.00
			Autosampler, day 5	5.20
		plasma	Freeze–thaw stability 1 cycle	−9.50
			Freeze–thaw stability 2 cycle	−20.10
			Freeze–thaw stability 3 cycle	−26.00
**HQC**	Meropenem	extract	Autosampler, day 1	8.60
			Autosampler, day 5	−3.40
		plasma	Freeze–thaw stability 1 cycle	−16.40
			Freeze–thaw stability 2 cycle	−24.30
			Freeze–thaw stability 3 cycle	−60.20
	Ceftazidime	extract	Autosampler, day 1	−7.80
			Autosampler, day 5	−12.40
		plasma	Freeze–thaw stability 1 cycle	−4.50
			Freeze–thaw stability 2 cycle	−7.90
			Freeze–thaw stability 3 cycle	−15.70
	Ceftolozane	extract	Autosampler, day 1	0.40
			Autosampler, day 5	−3.20
		plasma	Freeze–thaw stability 1 cycle	−1.40
			Freeze–thaw stability 2 cycle	−8.20
			Freeze–thaw stability 3 cycle	−16.30
	Tazobactam	extract	Autosampler, day 1	0.40
			Autosampler, day 5	−3.20
		plasma	Freeze–thaw stability 1 cycle	−1.50
			Freeze–thaw stability 2 cycle	−4.80
			Freeze–thaw stability 3 cycle	−12.90
	Cefiderocol	extract	Autosampler, day 1	−5.40
			Autosampler, day 5	−12.40
		plasma	Freeze–thaw stability 1 cycle	−14.30
			Freeze–thaw stability 2 cycle	−33.20
			Freeze–thaw stability 3 cycle	−73.60
	Ceftobiprole	extract	Autosampler, day 1	−5.60
			Autosampler, day 5	−8.40
		plasma	Freeze–thaw stability 1 cycle	−4.60
			Freeze–thaw stability 2 cycle	−7.30
			Freeze–thaw stability 3 cycle	−14.90
	Avibactam	extract	Autosampler, day 1	4.00
			Autosampler, day 5	4.80
		plasma	Freeze–thaw stability 1 cycle	−2.70
			Freeze–thaw stability 2 cycle	−4.20
			Freeze–thaw stability 3 cycle	−8.50
	Fosfomycin	extract	Autosampler, day 1	8.32
			Autosampler, day 5	5.20
		plasma	Freeze–thaw stability 1 cycle	−5.30
			Freeze–thaw stability 2 cycle	−4.90
			Freeze–thaw stability 3 cycle	−4.60
	Vaborbactam	extract	Autosampler, day 1	4.80
			Autosampler, day 5	−0.2
		plasma	Freeze–thaw stability 1 cycle	−9.80
			Freeze–thaw stability 2 cycle	−16.20
			Freeze–thaw stability 3 cycle	−26.70

## Data Availability

The data presented in this study are available on request from the corresponding author.

## Abbreviations

The following abbreviations are used in this manuscript:

TDM	Therapeutic drug monitoring
UPLC-qTOF-MS/MS	Ultraperformance Liquid Chromatography-quadrupole-Time-of-Flight-Mass Spectrometry
LC	Liquid chromatography
MRM	Multiple Reaction Monitoring
IS	Internal standard
EMA	European Medicine Agency
MRSA	Methicillin-resistant staphylococcus aureus
CE	Collision energy
DP	Declustering potential
QC	Quality control
LOQ	Limit of quantification
LQC	Low-quality control
MQC	Medium-quality control
HQC	High-quality control
CONC	Concentration
AVG	Average
S/N	Signal-to-noise ratio
CV	Coefficient of variation
ER	Extraction recovery
ME	Matrix effect
CI	Continuous infusion
